# Quantitative Insights and Visualization of Antimicrobial Tolerance in Mixed-Species Biofilms

**DOI:** 10.3390/biomedicines11102640

**Published:** 2023-09-26

**Authors:** Mandy Dittmer, Florian H. H. Brill, Andreas Kampe, Maria Geffken, Julian-Dario Rembe, Raphael Moll, Ifey Alio, Wolfgang R. Streit, Eike Sebastian Debus, Ralf Smeets, Ewa Klara Stuermer

**Affiliations:** 1 Department of Vascular Medicine, Translational Research, University Heart Center, University Medical Center Hamburg-Eppendorf, 20246 Hamburg, Germany; m.dittmer@uke.de (M.D.);; 2 Dr. Brill + Partner GmbH, Institute for Hygiene and Microbiology, 22339 Hamburg, Germany; 3 Institute for Transfusion Medicine, University Medical Center Hamburg-Eppendorf, 20251 Hamburg, Germany; 4 Department of Vascular and Endovascular Surgery, Heinrich-Heine-University of Düsseldorf, 40225 Düsseldorf, Germany; 5 Department of Microbiology and Biotechnology, University Hamburg, 20148 Hamburg, Germany; 6 Department of Oral and Maxillofacial Surgery, University Medical Center Hamburg-Eppendorf, 20246 Hamburg, Germany

**Keywords:** chronic wound, mixed-species biofilm, extrapolymeric substance (EPS), antimicrobials, antimicrobial dressings

## Abstract

Biofilms are a major problem in hard-to-heal wounds. Moreover, they are composed of different species and are often tolerant to antimicrobial agents. At the same time, interspecific synergy and/or competition occurs when some bacterial species clash. For this reason, the tolerance of two dual-species wound biofilm models of *Pseudomonas aeruginosa* and *Staphylococcus aureus* or *Enterococcus faecium* against antimicrobials and antimicrobial dressings were analyzed quantitatively and by confocal laser scanning microscopy (CLSM). The results were compared to findings with planktonic bacteria. Octenidine-dihydrochloride/phenoxyethanol and polyhexamethylene biguanide (PHMB) irrigation solutions showed a significant, albeit delayed reduction in biofilm bacteria, while the PHMB dressing was not able to induce this effect. However, the cadexomer-iodine dressing caused a sustained reduction in and killed almost all bacteria down to 10^2^ cfu/mL within 6 days compared to the control (10^10^ cfu/mL). By means of CLSM in untreated human biofilm models, it became evident that *P. aeruginosa* dominates over *E. faecium* and *S. aureus*. Additionally, *P. aeruginosa* appeared as a vast layer at the bottom of the samples, while *S. aureus* formed grape-like clusters. In the second model, the distribution was even clearer. Only a few *E. faecium* were visible, in contrast to the vast layer of *P. aeruginosa*. It seems that the different species avoid each other and seek their respective niches. These mixed-species biofilm models showed that efficacy and tolerance to antimicrobial substances are nearly species-independent. Their frequent application appears to be important. The bacterial wound biofilm remains a challenge in treatment and requires new, combined therapy options.

## 1. Introduction

Following the individualized therapy of the underlying disease, wound biofilm is the greatest local challenge in hard-to-heal wounds. About 75% of all chronic wounds are densely populated with biofilm [1,2]. Implants in vascular surgery or orthopedics are often colonized by biofilms consisting of multiple species [1,3]. The standardized analyses of wound swabs performed in routine clinical practice using nutrient agar and light microscopy usually detect only a very small proportion of biofilm-forming species in chronic wounds [4]. At present, it is still unclear if the diversity of species in the biofilm is important and to what extent this is clinically relevant in wound infection.

During the formation of wound biofilms, planktonic bacteria cells attach themselves to the wound bed and produce an extracellular matrix (EPS) consisting of lipids, polysaccharides, proteins and extracellular DNAs [5,6,7]. This helps the microorganisms to adhere strongly to the wound bed, making them difficult to remove [6].

Another important component of the EPS matrix is water. In this way, bacteria are protected from dehydration [6,7,8]. The dry mass of most biofilms consists of over 90% EPS matrix and only 10% microorganisms [6]. Due to biofilm formation, embedded bacteria are more tolerant against host immune defense and various types of antimicrobial treatments (e.g., antibiotics and ultraviolet radiation) [6,7]. The biofilm matrix serves as a fortress that protects the bacteria from contact with antimicrobial agents [6,7,9]. 

Furthermore, biofilms show a high diversity with synergetic interaction, such as cell–cell communication and horizontal gene transfer [6,7]. Especially, Quorum sensing is a kind of communication through which many activities, such as adhesion, biofilm formation, virulence factors, horizontal gene transfers and even antibiotic resistance, can be controlled [6,7,8,9,10]. The various factors increase resistance against antibiotics and other treatments. 

However, there are species, such as *P. aeruginosa*, which can produce antimicrobial agents against other bacteria. In this way, one bacterial species can dominate another in a mixed-species biofilm [11,12,13]. In many biofilm-burdened human wounds, where *P. aeruginosa* and other species could be detected in the microbiological wound swab analysis, a clinical dominance of *P. aeruginosa* is evident [14]. In addition to the typical greenish staining of the necrotic tissue and/or wound dressing, this can be detected by means of the typical cyan-blue glow of the pyoverdines (metabolic catabolites of *P. aeruginosa*) when exposed to UV–near light (405 nm; e.g., MolecuLight^®^, MolecuLight Corp., Toronto, ON, Canada) [15]. However, according to the manufacturer, this is only possible with a high bacterial density of more than 10^4^ cfu/g.

Independent of the bacterial coexistence, a possible symbiosis or competition in the human-wound biofilm of chronic wounds, the question arises as to whether, in a multispecies biofilm, bacteria exert a synergistic behavior becoming more tolerant to antimicrobials and wound dressings containing active ingredients. In addition, under this vital challenge, the dominance of one species over another could be enhanced, or circumstantial pressure might select species of lesser quantities to gain pathogenic potential. First, answers to this question are presented by the analysis of three of the most frequently detected bacterial species in chronic wounds [16,17] in combination with each other and under additional challenge with antimicrobial wound irrigation solutions and wound dressings frequently used in Europe. 

## 2. Methods

### 2.1. Test Organisms and Nutrients

The test organisms *Staphylococcus aureus* (ATCC 6538), *Pseudomonas aeruginosa* (ATCC 15442) and *Enterococcus faecium* (ATCC 6057) were chosen as common pathogens on chronic wounds and typical biofilm builders. Bacterial strains were cultivated on casein/soy peptone agar plates (CSA) according to EN 13727. The second subculture was used for testing. Each bacterial suspension was adjusted to a 0.5 McFarland standard (approx. 1.5 × 10^8^ cfu/mL) using a densitometer (Grant Bio™ DEN-1B, Grant Instruments Ltd.; Cambs SG8 6 GB, Cambridge, UK).

### 2.2. Preparation of the Leucocyte-Rich Human-Plasma Biofilm Model (lhBIOM)

The preparation of the lhBIOM was performed as described previously [18]. In brief, fresh frozen plasma (FFP; citrate-buffered) and one LRS^®^ chamber of leukocyte apheresis (Trima Accel^®^; Terumo BCT Inc.; Lakewood, CO, USA) were obtained from the Institute for Transfusion Medicine (University Medical Center Hamburg-Eppendorf, Hamburg, Germany). All donors provided their informed and written consent for the use of their blood products. FFP was thawed, adjusted to 250 mL and the “immunocompetence” of a platelet donor was added. The immune cells (about 40 × 10^3^ leukocytes/µL) were harvested by using a special automated blood collection system for apheresis Terumo BCT design (Trima Accel^®^ LRS^®^ Platelet, Plasma Set, REF number 82,300; Terumo BCT Inc., Lakewood, CO, USA). The content of the LRS^®^ chamber was placed in a tube and centrifuged at 1610× *g*. The remaining erythrocytes were gently removed and the plasma–leukocyte mix was added to the FFP. After gentle mixing, the combined bacterial suspension (*P. aeruginosa* and *S. aureus* or *P. aeruginosa* and *E. faecium*) was added, resulting in a final concentration of 2.5 × 10^7^ cells/mL (1.5 × 10^6^ cfu/model). Next, 18.26 µL of CaCl_2_ (500 mM) per mL plasma was added to the bacteria–plasma mix to induce coagulation. The resulting biofilm mixture was immediately transferred into 12-well plates (1.5 mL per model/well). The well plates were placed in a rotation shaker and incubated for 18 h at 60 rpm and 37.0 °C to polymerize and form an extracellular matrix. 

### 2.3. Antimicrobial Treatment of the Biofilm Models and Quantification of the Bacterial Load

Two antiseptic and antimicrobial irrigation solutions were used with the active agent octenidine-dihydrochloride/phenoxyethanol (0.1% OCT/2% PE: Schülke&Mayr; Norderstedt, Germany) and polyhexamethylene biguanide (0.1% PHMB, B. Braun; Melsungen, Germany), respectively. The agents were used in commercially available concentrations and compared to the untreated controls. Each biofilm disc was treated with 300 µL of the solution for 24, 48 and 72 h, with repeated applications of 300 µL every 24 h. After the specified treatment periods, the antimicrobial activity was terminated by adding an equivalent volume of the neutralizing solution TLSNt-SDS (6% polysorbate 80, 6% saponin, 0.8% lecithin, 2% sodium dodecyl sulphate and 0.6% sodium thiosulphate in di-water) to each well for 5 min at room temperature in a rotation shaker. 

For testing the performance of antimicrobial dressings on mixed-species biofilms, a polyhexanide-containing dressing (0.25–0.65 mg/cm^2^ PHMB; Suprasorb^®^ P, Lohmann & Rauscher GmbH, Vienna, Austria), a cadexomer-iodine-containing (0.90% *w*/*w* C-IOD; Iodosorb^®^, Smith&Nephew GmbH, London, UK) and an agent-free control dressing (Urgo Clean, Urgo GmbH; Sulzbach, Germany) were used. All dressings were prepared in an aseptic manner with a diameter of 2.2 cm (A = 3.8 cm^2^), fitting press-fit, in one well of a standard 12-well plate (Sarstedt, Nuembrecht, Germany) containing an lhBIOM. Additionally, weighted with 20 g, a wound-like scenario with direct dressing–biofilm contact was created. The dressings remained on the biofilm without change for 1, 3 and 6 days and were surrounded by fluid (FFP, immune cells and bacterial products). Plastic beakers (50 mL) were filled with glass beads (D = 3–4 mm) covering the bottom, and 10 mL of the neutralizer solution TLSNt-SDS (6% polysorbate 80, 6% saponin, 0.8% lecithin, 2% sodium dodecyl sulfate and 0.6% sodium thiosulfate in aqua dest.) was added. The dressings were placed in this solution and shaked for 10 min at 200 rpm, and the extracts thus obtained were plated out in tenfold dilutions on CSA agar. After incubation at 37 °C under aerobic conditions for 48 h, the quantification of colony-forming units using a digital colony counter (NSCA 436000, VWR International GmbH; Darmstadt, Germany) was performed.

The antimicrobial activity in the lhBIOM was neutralized by adding 300 µL of the neutralizing solution TLSNt-SDS (see above) to each well. The plates were subsequently placed on a rotation shaker for 5 min at room temperature for the incubation of neutralizing agents. Biofilm models independent of the treatment were then dissolved by adding 3 mL (1:1 *v*/*v*) 10% (*w*/*v*) bromelain (Bromelain-POS^®^, Ursupharm Arzneimittel; Saarbrücken, Germany) after detachment from the wall of the well and puncturing with a pipette tip for several times to facilitate the bromelain distribution for dissolving the model. For quantification, the resulting solution was serially diluted tenfold. A total of 50 µL of each dilution was plated on CSA (spread technique) and incubated for 48 h at 37 °C under aerobic conditions. The colony-forming units (in cfu/mL) were determined using a manual colony counter (Schuett count, Schuett-Biotec GmbH; Göttingen, Germany). 

### 2.4. Quantitative Suspension Method (QSM)

For addressing differences in bacterial response to antimicrobials in planktonic form and biofilm formation, mixed strains were also evaluated in a quantitative suspension method (QSM) based on DIN EN 13727 with a high organic load (3.0 g/L bovine albumin + 3.0 mL/L sheep erythrocytes, i.e., “dirty conditions”). The bacterial test suspensions of *P. aeruginosa* and *S. aureus* (M1) and *P. aeruginosa* and *E. faecium* (M2) were adjusted in a sum of 1.5 × 10^8^ cfu/mL (0.5 McFarland standard) initial concentration in tryptone salt broth. OCT/PE and PHMB solutions were added to the concentrated DMEM, yielding final test concentrations of 80%, 50% and 10% (*v*/*v*). For testing the antimicrobial activity, 8 mL of the different suspensions and 1 mL of the organic load were exposed to 1 mL of the test solution for 60 s. For the neutralization of actives, 1 mL of the test suspension was transferred to 8 mL of the neutralizer containing 60 g/L of polysorbate 80, 60 g/L of saponine, 8 g/L of lecithin, 1 g/L of histidine, 2.5 g/L of SDS and 1 mL of di-water. The neutralization time was 10 s. The surviving bacteria (in cfu/mL) were quantified on agar plates as described for the biofilm models.

### 2.5. Brill–Braunwarth Method

For evaluating the antimicrobial efficacy of wound dressings against planktonic cells, the Brill–Braunwarth method was used [19,20]. Agar plates were inoculated with 0.1 mL suspension of test organisms. As described above, two mixed inoculates were used with initial cell counts of 3–10 × 10^6^ cfu. Pre-wetted test samples were applied to the agar surface and weighed down for full contact. After 24 h the agar underneath the samples was cut out. The cut-out agar was transferred into a validated neutralizer suspension (1 g/L polysorbate 80 + 1 g/L sodium thiosulfate) in a sterile masticator bag. The suspension was homogenized using a masticator and incubated at room temperature for 5 min. Colony-forming units were analyzed by plating dilutions from the suspension treated in the masticator on TSA.

### 2.6. Statistical Analysis

Values were expressed as mean ± standard error of the mean (MV ± SEM) with a 95% confidence interval (CI) based on triplicates, derived of three different anonymous blood donors regarding the lhBIOM. Bacterial reduction rates (in Δlog_10_ cfu/mL) were calculated using GraphPad PRISM (Version 8.2.1; GraphPad Software Inc., La Jolla, CA, USA). Statistical analysis contained a two-way ANOVA and followed by Holm–Sidak post hoc test for the evaluation of multiple comparisons. A *p*-value of ≤0.05 was considered statistically significant. The histological analysis was descriptive and qualitative without statistical considerations.

### 2.7. Microscopic Imaging of the Mixed Biofilm Models with and without Antimicrobial Treatment

In 8-well Ibidi chamber slides (Ibidi GmbH; Gräfelfing, Germany), the biofilm model was prepared for the laser scanning microscope (LSM 800 Carl Zeiss AG, Oberkochen, Germany) in combination with the C-Apochromat objective (63×/1.20 W) (Carl Zeiss Microscopy GmbH; Hamburg, Germany) with immersion oil. For this investigation, labelled microorganisms were used (*P. aeruginosa* (PAO1) mCherry, *S. aureus* (SH1000) and *E. faecium* (BSU 385 kindly provided by B. Spellerberg, Institute of Medical Microbiology; University of Ulm; Ulm, Germany [21]) GFP). First, 300 µL of the biofilm mixture was transferred into each well of the Ibidi chamber. After incubation at 37 °C, each chamber (except for the control) was treated with 60 µL OCT/PE or PHMB. The experiment was performed on a slide, treated after 24, 48 and 72 h with OCT/PE or PHMB. In addition, a control without treatment was run. 

Following image capturing, the slides were re-incubated and further treated with a repetitive application as described above for subsequent imaging time-points. Therefore, no neutralization was performed.

Three-dimensional reconstructions and mosaic images, which consisted of 20 single shots, were created using the software ZEN (version 2.3; Carl Zeiss Microscopy GmbH; Hamburg, Germany).

## 3. Results

### 3.1. Efficacy of Wound Irrigation Solutions on Planktonic Bacteria Cells

The results in this section present a first overview of the efficacy of wound irrigation solutions over the bacteria strains based on planktonic cells. After treatment with commercially available OCT/PE and PHMB wound irrigation solutions in 10%, 50% and 80% concentrations, the bacterial count was determined. OCT/PE kills all bacterial species in 50% and 80% solutions. Only *P. aeruginosa* showed a growth ranging from 3.57 to 4.85 log_10_ cfu at a concentration of 10%, while the other bacteria were eradicated regardless of the combination. However, against *P. aeruginosa* at 10% OCT solution, lower reduction factors were obtained in combination with *S. aureus* than with *E. faecium*. For M1, the reduction in *P. aeruginosa* was around 64% in contrast to M2 with 50%. However, a complete bacterial eradication was shown in all species from a 50% concentration of the clinically used OCT/PE irrigation solutions onwards (Table 1).

PHMB also acted as bactericidal (reduction factor ≥ 5 log, according to EN 13727) at a concentration starting at a 50% dilution of its clinical irrigation solution. At 10% PHMB, the bacteria strains showed a growth ranging from 2.66 to 3.98 log_10_ cfu. Likewise, the bacterial composition was hardly different. Except for *S. aureus*, all bacteria had a log_10_ reduction of less than 4 log_10_, while the log_10_ reduction in this germ was 4.96 log_10_ (Table 1). 

### 3.2. Efficacy of the Antimicrobial Wound Dressings on Planktonic Cells

In contrast to the wound irrigation solutions, the efficacy of antimicrobial wound dressings was restricted. Primarily, C-IODINE showed a strong bactericidal effect, so that no surviving bacteria (cfu) could be counted while the bacterial count with the PHMB wound dressing was almost identical to the agent-free control dressing. Especially, in the case of *S. aureus*, around 10^8^ cfu/mL were counted after treatment with the PHMB dressing, while less than 10^8^ cfu/mL were detected in the control (Table 2). 

### 3.3. Antimicrobial Efficacy of the Wound Irrigation Solution on the Different Mixed-Species Biofilms

**Figure 1:**
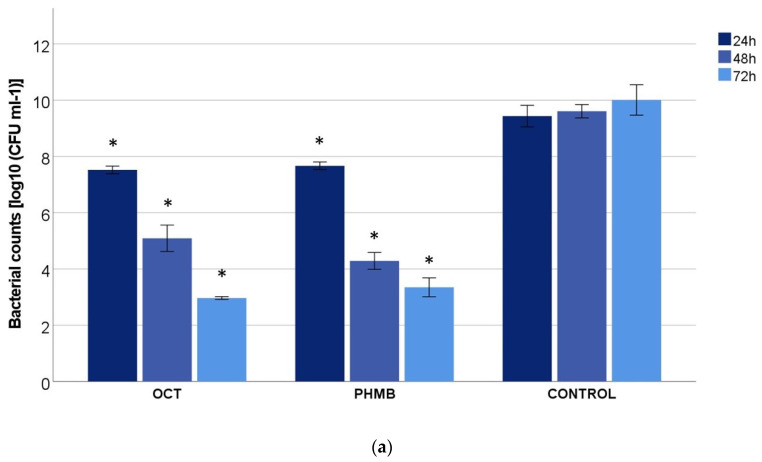

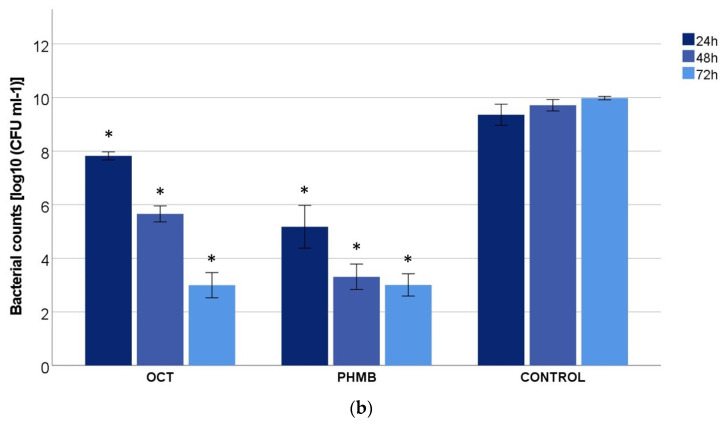
Bacterial counts of (**a**) M1 (*P. aeruginosa* + *S. aureus*) and (**b**) M2 (*P. aeruginosa* + *E. faecium*) after treatment with the wound irrigation solutions octenidine-dihydrochloride/phenoxyethanol (OCT/PE) and polyhexamethylene biguanide (PHMB) and the control after 24, 48 and 72 h (values expressed as mean ± SEM; * *p* ≤ 0.05 vs. the control).

The analysis of the two wound irrigation solutions showed nearly similar results for the mixed-species biofilm models M1 and M2. While the bacterial counts of the control slightly increased from 24 to 72 h, the wound irrigation solutions OCT/PE and PHMB induced a significant decrease down to 5 log steps, which by definition corresponds to a bactericidal effect (Figure 1). Already after one day, a reduction of >1 log_10_ by OCT/PE and 1–4 log_10_ by PHMB was seen, in which PHMB had a stronger effect on *P. aeruginosa* and *E. faecium* than on the mixed biofilm with *P. aeruginosa* and *S. aureus*. Treatment with OCT/PE showed only slight differences between the models. After 72 h, the bacterial counts between the different models treated with OCT/PE or PHMB were nearly the same, with approx. 10^3^ cfu/mL in contrast to the control, with approx. 10^10^ cfu/mL.

### 3.4. Anti-Biofilm Activity of the Antimicrobial Wound Dressings in the Different Mixed-Species lhBIOM

**Figure 2:**
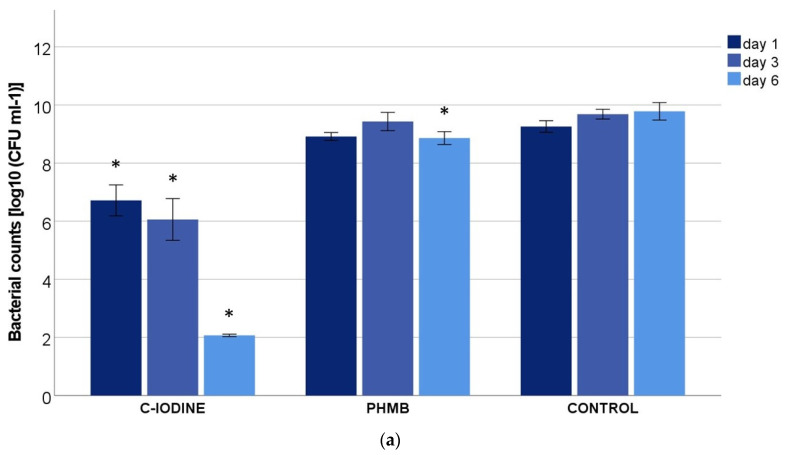

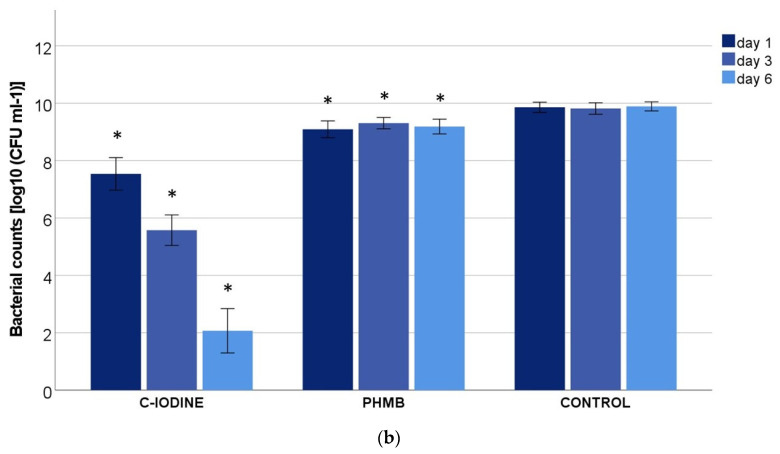
Bacterial counts of (**a**) M1 (*P. aeruginosa + S. aureus*) and (**b**) M2 (*P. aeruginosa* + *E. faecium*) after treatment with the antimicrobial dressings containing cadexomer-iodine (C-IODINE) or polyhexamethylene biguanide (PHMB) and the control after 1, 3 and 6 days (values expressed as MV ± SEM * *p* ≤ 0.05 vs. the control).

In contrast to the wound irrigation solutions, the two antimicrobial dressings performed differently, as the reduction in the colonies in the PHMB dressing was almost not significant in contrast to the control. After one day of treatment of the M2 biofilms, the bacterial growth was only one log_10_ reduced compared to the agent-free dressing (Figure 2a), with no further reduction on days 3 and 6. In M1 biofilms with the combination of *P. aeruginosa* and *E. faecium*, a trend reduction of 1–2 log_10_ could be observed only after 6 days. The C-IODINE dressing reduced the bacteria in both models M1 and M2 by 2–3 log_10_ after one day (Figure 2); unfolded without dressing changed its effect further, so that, on day six, only about 2 log_10_ of bacteria remained. 

### 3.5. Quantitative Microbial Load in Wound Dressings

**Figure 3:**
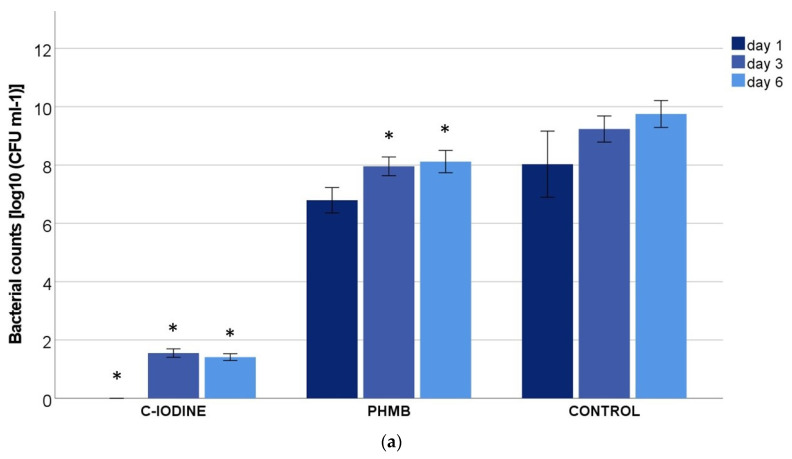

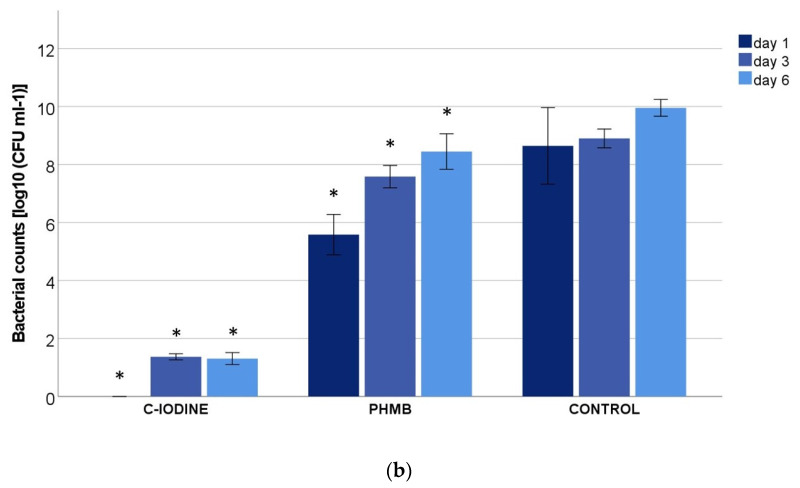
Bacterial counts in (**a**) M1 *(P. aeruginosa* + *S. aureus)* and (**b**) M2 *(P. aeruginosa* + *E. faecium)* in exudate equivalents in the wound dressings containing cadexomer-iodine (C-IODINE), polyhexamethylene biguanide (PHMB) or no active ingredients (control) after 1, 3 and 6 days of incubation (values expressed as mean ± SEM; * *p* ≤ 0.05 vs. the control).

While the results shown in Figure 2 refer translationally to the possible reduction in bacteria in the wound, Figure 3a,b show the bacterial uptake in the exudate equivalent of the dressings. In the agent-free control dressing, an almost constant bacterial content of 8–10 log_10_ was detected in the eluate. The PHMB dressing also showed a constant, M1- and M2-independent bacterial content of 6–8 log_10_. In the eluate of the C-IODINE dressing, no bacteria were counted after day one. Even after 3 or 6 days, not even 100 cfu/mL were counted (Figure 3a,b), showing that there is no significant difference between M1 and M2. However, it is significantly different from the PHMB-containing and agent-free control dressings.

### 3.6. Microscopy Imaging of the Bacterial Strains in the lhBIOM

**Figure 4:**
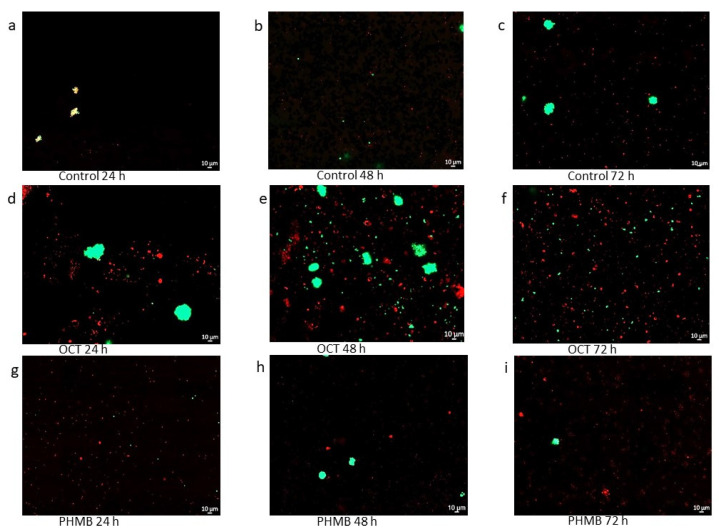
Confocal laser scanning microscopy (CLSM) mosaic images consisting of 20 single shots of the mixed-species lhBIOM consisting of M1 (*P. aeruginosa* (mCherry) + *S. aureus* (GFP)) as (**a**–**c**) the untreated control and (**d**–**f**) 24, 48 and 72 h after the application of the wound irrigation solutions containing octenidine-dihydrochloride/phenoxyethanol (OCT/PE) or (**g**–**i**) (polyhexamethylene biguanide (PHMB) (63× magnification).

**Figure 5:**
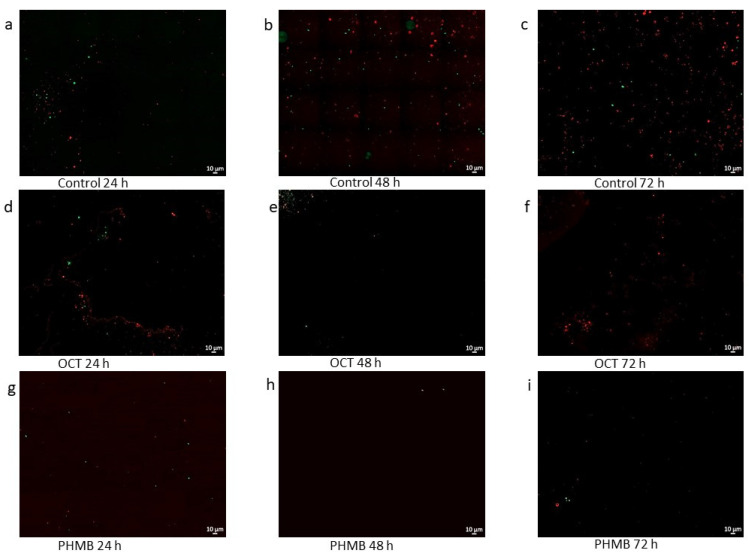
Confocal laser scanning microscopy (CLSM) mosaic images consisting of 20 single shots of the mixed-species lhBIOM consisting of M2 (*P. aeruginosa* (mCherry) + *E. faecium*(GFP)) as (**a**–**c**) the untreated control and (**d**–**f**) 24, 48 and 72 h after the application of the wound irrigation solutions containing octenidine-dihydrochloride/phenoxyethanol (OCT/PE) or (**g**–**i**) (polyhexamethylene biguanide (PHMB) (63× magnification).

With the help of a confocal microscopy, the ratio between the species in the multispecies biofilm became visible (Figure 4 and Figure 5). The bacteria from M1 had a different appearance, while *S. aureus* had an aggregated structure; *P. aeruginosa* was widely distributed. 

In contrast to the control, it seems that *S. aureus* initially survives better than *P. aeruginosa* in the sample with the OCT/PE treatment. In this case, the difference between the species also became much more evident. However, a slight decline in the bacteria became apparent (Figure 4).

**Figure 6:**
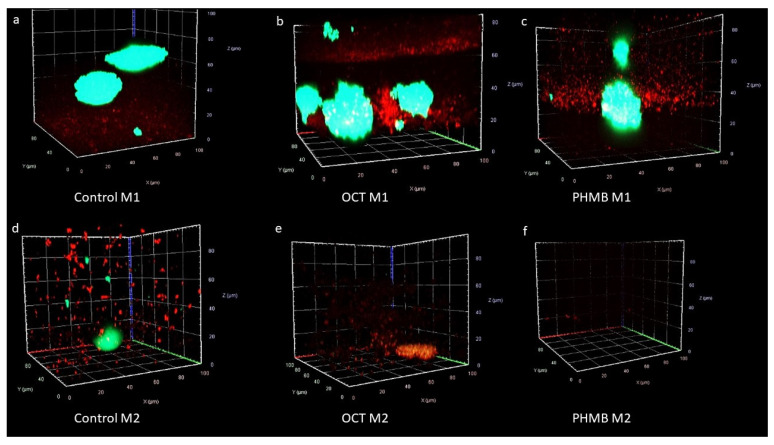
Visualization of the bacterial configuration in the lhBIOM of the mixed-species (**a**–**c**) M1 (*P. aeruginosa* (mCherry) + *S. aureus* (GFP)) and (**d**–**f**) M2 (*P. aeruginosa* (mCherry) + *E. faecium* (GFP)) by 3D confocal laser scanning microscopy (CLSM) after 48 h with and without the application of wound irrigation solutions containing octenidine-dihydrochloride/phenoxyethanol (OCT/PE) or polyhexamethylene biguanide (PHMB) (63× magnification).

The PHMB sample was similar to the control (Figure 4) but also with a slight decrease in bacteria. In addition, in some images, the platelets of the lhBIOM fluoresced red (Figure 4e,i). In the 3D images, it seems that *P. aeruginosa* populated the lower layers of the sample, while *S. aureus* stayed true to their name and formed big grape-like clusters (Figure 6). 

In contrast to M1, the bacteria of M2 were not different in their appearance. In this multispecies sample, it became visible that *E. faecium* was less present than *P. aeruginosa* (Figure 5). The 3D images of the control also showed uniform layers of *P. aeruginosa* at the bottom. *E. faecium* showed no uniform layer but was ubiquitously present (Figure 6). In the control, *E. faecium* clustered in the higher layers. However, after 48 and 72 h, the strain was hardly visible (Figure 5 and Figure 6). More than in M1, the decrease in the bacterial strains after treatment with OCT/PE or PHMB became visible (Figure 5 and Figure 6). 

## 4. Discussion

Translational research provides an essential contribution to closing the gap between basic research and clinical reality. It is evident that the human environment plays a significant role in the expression of an effect and, in particular, makes a difference in the quality and quantity of the biofilm [22]. A wound exudate originates in blood serum, and thus the protein content and composition of an acute wound exudate and serum show little difference [23,24]. 

However, depending on the degree of healing, the wound exudate also has a different quantitative composition with regard to growth factors or bacterial metabolites and toxins. For example, interleukin-1β and matrix metalloproteases (MMP) 2 [25,26] or lactate [27] increase with inflammation and infections. The wound environment, represented in lhBIOM by human blood plasma, immune cells, bacteria themselves and their products, also influences the composition of the EPS of the biofilm [14]. This plays an essential role in the biofilm’s typical tolerance to antimicrobials [22]. It is a mechanical, chemical and physical barrier. The EPS with its embedded and surrounding bacteria also interacts with the human cells of the wound bed [14], which in turn causes changes in the environment. Biofilm models free of human components do not allow such deep insights into host–bacteria interactions [28,29]. Despite this knowledge, most analyses concerning the topic of “wound biofilm” are based on the growth of bacteria on adhesive materials (plastic wells, glass slides and medical devices) [30,31] or three-dimensional in vitro models based on a liquid-filled chamber [30,32] or are comprised of a collagen matrix with serum proteins [33].

Another factor that influences the biofilm, its structure and matrix, is the bacteria that interact with it and build it up. It is known that, in multi-species biofilms typical of chronic wounds, bacteria interact synergistically and competitively with each other [22]. 

In this study, mixed-species lhBIOMs containing *P. aeruginosa*, *S. aureus* and *E. faecium* were chosen, because they belong to the most common bacterial pathogens isolated from chronic wound infections [34,35]. In this paper, the laser scanning microcopy images showed a dominance of *P. aeruginosa* over *S. aureus* but one that was higher over *E. faecium* (Figure 4 and Figure 5). At the same time, it became apparent that the species avoid each other; so, the samples did not have a homogeneous bacterial distribution. Especially, *S aureus* formed staphylococci clusters to escape from *P. aeruginosa* (Figure 4 and Figure 6). A possible reason for the different growth of the bacteria in the lhBIOM could be the generation time of the individual bacteria. Even when the doubling times are similar under optimal conditions, the medium does not fulfil the requirements of the bacteria to the same extent [36,37,38]. Different generation times could lead to the faster growing species being able to colonize the surfaces, thus limiting the resources for the slower growing species and thus their survival [39]. In the model with *E. faecium* and *P. aeruginosa*, the different growths could be due to the low presence of *E. faecium*; so, *P. aeruginosa* has already taken over the resources before *E. faecium* could spread. Nevertheless, a complicating factor was the autofluorescence of some red blood cells. In further studies, a possible suppression would be appropriate to distinguish the human components from the bacterial ones [40]. 

Looking at the numerical distribution of bacteria over time, it is likely that *P. aeruginosa* displaced a large proportion of *S. aureus* and thus prevented the orchestrated biofilm build-up of this competing species [25]. There are known antimicrobial systems of *P. aeruginosa* against *S. aureus*, such as iron-regulated antimicrobial activity because of multiple alkylquinolones by cystic fibrosis isolates [12], the secretion of proteases that can lyse *S. aureus* [11] or the production of pyocyanin that inhibits the growth of *S. aureus* [41]. Similar processes may have led to the decrease in the number of *S. aureus* cells on the images and the planktonic cell assays. 

Thus, it is reasonable to assume that the response of the biofilm to antibiotics and antiseptics is also dominated by *P. aeruginosa*. Studies reported a co-existent or niche formation of *P. aeruginosa* together with *S. aureus*. Furthermore, in chronic wounds, which were contaminated with both species, *P. aeruginosa* was located in deeper regions of the wound than *S. aureus* [35,42]. This was also visible in the 3D images, where *P. aeruginosa* tended to colonize the lower layers and *S. aureus* the upper layers. The extent to which *S. aureus* is relevant to *P. aeruginosa* or possibly even beneficial in the mixed-species biofilm is still unclear. Overall, however, it has been proven that a multispecies biofilm develops higher tolerance and causes a greater tissue infiltration [43]. 

In clinical wounds, this is apparent, but also not proven. On the other hand, in the microbiological results of swabbing, if *Pseudomonas* is present, its numerical dominance is usually observed (unpublished data). Using UV–near light (405 nm, MolecuLight^®^, MolecuLight Corp.; Toronto, ON, Canada) to detect wound areas with a high bacterial burden (10^4^ cfu/g) also reflects this phenomenon: when *P. aeruginosa*-specific metabolic products (e.g., pyoverdins) are detected by means of cyan blue fluorescence, a wide distribution and a bright glow are very often observed due to a high level of colonization and infiltration of the wound. The rather unspecific red fluorescence of the metabolic products (e.g., porphyrins) of, e.g., Gram-positive *S. aureus* or Enterobacteria, is considerably inferior here [14]. However, if *P. aeruginosa* is not detectable among the wound pathogens, the red fluorescence is much more pronounced and widespread. The clinical picture here exhibits a visual correlation to confocal laser scanning microscope images (Figure 6a). 

Considering the analyses of the antimicrobial wound irrigation solutions and wound dressings, the aforementioned increase in antiseptic tolerance for the tested mixed-species biofilms with *P. aeruginosa* and *S. aureus* and *E. faecium*, respectively, cannot be reproduced. Comparing the antimicrobial effects of OCT/PE and PHMB (limited in principle in biofilms) with the results of the respective single-species biofilms in the same human model [18,44,45], there were no differences apparent. In the *P. aeruginosa* single-species biofilm, the PHMB wound dressing produced a sustained reduction of 2–3 log steps over the test period of 72 h; in both mixed-species biofilms, the reduction was 1–2 log steps; so, there is no apparent evidence of an increase in the tolerance of the tested species through interaction. It seems evident that the biofilm matrix (EPS), which is mainly composed of polysaccharides and proteins, inhibits PHMB from penetrating deeper and damaging the bacterial membrane and DNA [46]. Recent in vitro, in vivo and human studies also showed that the agent polyhexamethylene guanidine (PHMG), chemically quite similar to PHMB, appears to have a stronger potential to attack wound biofilms, and clinical results are promising in terms of bacterial reduction [47,48]. The role of additive wound debridement should certainly not be underestimated.

The antibacterial effect in the highly effective C-IODINE-containing wound dressing also shows only small differences between single- [49] and mixed-species biofilms. These observations have to be verified in a study with single and mixed species, so that the results can be compared directly. In addition, the possible effect of mixed-species biofilms and interspecies interaction remains to be investigated, e.g., by transcriptome analysis, selective cultivation/PCR quantification or side-by-side comparison of single-species and mixed-species biofilms. Nevertheless, a decrease in the number of germs was observed for both multispecies over time with OCT/PE and PHMB, which was visible in the microscopy images too (Figure 4 and Figure 5). 

Comparing the results of the mixed-species lhBIOMs with the planktonic mixed cultures of the same species, the high pre-described efficacy [50,51] of OCT/PE and PHMB in planktonic settings was proven. Only a 10% solution of the wound irrigation solution used in clinical practice allowed for the growth of *P. aeruginosa* (OCT/PE and PHMB) and *E. faecium* (PHMB). As described previously [52], the PHMB-releasing wound dressing also shows a loss of efficacy in the planktonic mixed cultures, presumably due to the high protein load and comparably low overall concentration of the PHMB agent within the dressing. This is independent of the respective bacterial species. In contrast, the wound dressing containing C-IODINE showed a relevant and significant reduction in all bacteria in the mixed cultures during the test period. However, it cannot be concluded from the present results whether the PHMB-releasing dressing would develop its antiseptic efficacy with a lower protein load.

As expected, the 3D CLSM images of the treated models showed a decrease in bacterial cells over time and repeated treatment. Conspicuous is the strong presence of *S. aureus*. Possibly, *P. aeruginosa* have to protect themselves against antimicrobials rather than fight against foreign strains. In addition, the grape cluster of *S. aureus* could be more protective to save the bacteria inside the formation than *P. aeruginosa*, which provide a large surface for attack. On the other hand, there is research that addresses the opportunistic effect of antimicrobial substances of *P. aeruginosa*, which can increase antibiotic tolerance in *S. aureus* due to the inhibition of respiration and the depletion of intracellular ATP [53]. The question of whether exoproducts of *P. aeruginosa* promote the antimicrobial tolerance of *S. aureus* is still open. Further research could improve the understanding of interspecific interactions and provide information on tolerance to antimicrobials to improve the treatment and healing of infected wounds.

## 5. Conclusions

In order to understand the formation and tolerance to antimicrobials in wound biofilms, 3D models are required that incorporate as many components as possible of the patient’s own, i.e., human, wound biofilm; this occurs in the lhBIOM. In particular, the 3D CLSM showed a very interesting distribution of *S. aureus* clusters in the presence of *P. aeruginosa*—suggested to be based on interspecies competition. On the other hand, *E. faecium* did not survive the combination of *P. aeruginosa* and the antimicrobial treatment. Further analyses are needed to decipher the population-specific behavior within multi-species biofilms, their relevance and the molecular mechanisms (e.g., quorum sensing) that explain the behavior of the species tested in this paper. In particular, how the interspecific interaction behaves in combination with antimicrobials. Moreover, the comparatively low antimicrobial efficacy of the tested antimicrobial dressings on biofilm bacteria is noteworthy. These observations support the need for wound bed preparation or debridement prior to their application in clinical practice. 

## Figures and Tables

**Table 1:** Log_10_ reduction in mixed-species suspensions (initial cell count: >10^7^ bacteria) after the application of octenidine-dihydrochloride/phenoxyethanol (OCT/PE) or polyhexamethylene biguanide (PHMB) solutions under dirty conditions. Analyses were based on DIN EN 13727:2015, whereas a log_10_ reduction ≥ 5 refers to a sufficient antibacterial efficacy.

Concentration	M1 lgN0 7.62				M2 lgN0: 7.20			
	OCT/PE		PHMB		OCT/PE		PHMB	
	*P. aeruginosa*	*S. aureus*	*P. aeruginosa*	*S. aureus*	*P. aeruginosa*	*E. faecium*	*P. aeruginosa*	*E. faecium*
10%	2.77	≥5.47	3.64	4.96	3.63	≥5.05	3.38	3.89
50%	≥5.47	≥5.47	≥5.47	≥5.47	≥5.05	≥5.05	≥5.05	≥5.05
80%	≥5.47	≥5.47	≥5.47	≥5.47	≥5.05	≥5.05	≥5.05	≥5.05

**Table 2:** Colony count (log_10_) in mixed-species suspensions (initial cell count (i.c.): >10^8^ bacteria) after the application of dressings containing cadexomer-iodine (C-IODINE) or polyhexamethylene biguanide (PHMB). Analyses according to the Brill–Braunwarth method were based on DIN 58940. The test was conducted in triplicate. M1: multispecies suspension with *P. aeruginosa* and *S. aureus*; M2: multispecies suspension with *P. aeruginosa* and *E. faecium* (MV: mean value; SEM: standard error of the mean).

Biofilm Model	Species	Control		PHMB Dressing		Cardexomer-Iodine Dressing	
		MV	SEM	MV	SEM	MV	SEM
M1 i.c. log 8.36 cfu/mL	*P. aeruginosa*	9.12	0.086	9.02	0.096	0	0
	*S. aureus*	7.59	0.419	8.41	0.014	0	0
M2 i.c. log 8.20 cfu/mL	*P. aeruginosa*	7.31	0.086	7.54	0.083	0	0
	*E. faecium*	6.23	0.17	6.35	0.071	0	0

## Data Availability

The data presented in this study are available upon request from the corresponding author.
